# Genomer — A Swiss Army Knife for Genome Scaffolding

**DOI:** 10.1371/journal.pone.0066922

**Published:** 2013-06-24

**Authors:** Michael D. Barton, Hazel A. Barton

**Affiliations:** Biology Department, The University of Akron, Akron, Ohio, United States of America; J. Craig Venter Institute, United States of America

## Abstract

The increasing accessibility and reduced costs of sequencing has made genome analysis accessible to more and more researchers. Yet there remains a steep learning curve in the subsequent computational steps required to process raw reads into a database-deposited genome sequence. Here we describe “Genomer,” a tool to simplify the manual tasks of finishing and uploading a genome sequence to a database. Genomer can format a genome scaffold into the common files required for submission to GenBank. This software also simplifies updating a genome scaffold by allowing a human-readable YAML format file to be edited instead of large sequence files. Genomer is written as a command line tool and is an effort to make the manual process of genome scaffolding more robust and reproducible. Extensive documentation and video tutorials are available at http://next.gs.

## Introduction

The decreasing costs and increasing diversity of high-throughput sequencing methods is making genome analysis a common method to tackle unresolved questions in microbiology [1]. Sequencing can produce 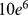
–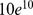 short nucleotide reads, which must be assembled into larger contigs that are scaffolded into larger megabase fragments or complete chromosomes. These larger DNA sequences are then annotated with genomic features such as protein-coding genes. However each of these required steps can produce imperfect results and remains the subject of active research [2]–[4]. Therefore manual curation of a genome sequence and corresponding annotations may still improve upon the results of automated methods.

Manually editing a genome sequence or annotation is non-trivial and requires effort on a researcher’s part. The large size of FASTA sequence files or general feature format (GFF) annotation files makes changing these in a text editor difficult. Tasks such as organising project files, adding additional sequences to an assembly, updating incorrect annotations or preparing files for database deposition all divert effort from the main goal of analysing a genome. As a result there is a steep learning curve for researchers embarking on genome sequencing for the first time, while still requiring effort from experienced researchers.

We anticipate these problems have resulted in individual groups independently producing in-house programming scripts to automate repetitive tasks. An example of such tasks include finding protein names that do not match a convention of three lower case characters followed by one upper case character (abcD). Such tasks are manually laborious but simple to automate computationally. Individual research groups producing their own scripts for such tasks however leads to a repetition of effort. In contrast open-source projects can lead to a pooling of community effort resulting in a higher standard of software and prevents reinvention of the same code [5].

During the process of sequencing several *Pseudomonas fluorescens* strains we developed a tool, called “Genomer,” to ease and simplify many of the common steps in our genome project. Several graphical user interface tools are available to work with genome projects, including G-InforBio, Genquire, Pile-LineGUI, Artemis and Consed [6]–[10]. Genomer instead provides a simple command line interface which allows scriptable automation and reproducible results. We made this software open-source under the MIT license and here we describe its application.

## Implementation

Genomer is written in the Ruby programming language (1.8.7 and 1.9.3) [11], [12] and built upon the Rubygems and Bundler Ruby package management libraries. Rubygems is a software management system for Ruby which allows automatic downloading and installation of third-party software libraries from the rubygems.org website. Bundler is software for determining which specific versions of the available third libraries should be used.

Genomer uses these two libraries to create a plugin system where third-party software can be written and included. Plugin creators need only to prefix the name of their plugin with genomer-plugin- and upload this to rubygems.org or make the plugin available as a public git repository. A plugin is specified within a Genomer project in a file named ‘Gemfile.’ The bundle update command will then automatically download and install the required plugins plus any gem dependencies these gems rely on.

Genomer is implemented as a command line tool and tested on both Mac OS X and Linux systems. The latest version of Genomer can installed from the Rubygems package management system with the command gem install genomer. Genomer is based on our previously described “Scaffolder” software [13] and is invoked on the command line with the command genomer. Genomer provides extensive documentation via UNIX manual pages and on the Genomer website at http://next.gs.

## Results and Discussion

Genomer was developed during our *P. fluorescens* genome projects to automate and simplify the manual steps required when finishing a microbial genome. Genomer provides the following functionality to facilitate this:

### Simple Editing of a Draft Genome Sequence

Genomer is built on the existing Scaffolder [13] file format for assembling draft genome sequences. This is format requires only the order and IDs of each contig be specified. This thereby simplifies the process of re-organising and trimming contigs in a draft genome without having to edit large nucleotide sequences manually.

### Mapping of Annotations onto the Assembled Sequence

Genomer maps the coordinates of contig annotations to their respective positions in the draft genome sequence. This allows the scaffold file to be continuously improved and updated even after the contigs have been annotated. For instance, additional PCR sequences can be used to close gaps in the assembly and the location of all annotations will be correspondingly updated.

### Generation of Files for Submission to GenBank

Submitting a genome sequence to GenBank database requires generating specific files. These may include a FASTA file of the draft sequence, a table of annotations, a FASTA file containing the individual contig sequences, and ‘a golden path’ (AGP) [14] file describing the placement of contigs. Genomer automates the generation of all these files from the scaffold file and corresponding GFF3 file thereby removing the effort to produce these manually.

### A Stable Interface and Streamlined Install Process

Genomer provides a plugin system built using the existing Ruby package management system: RubyGems and Bundler. This allows Genomer and its plugins to be automatically installed without requiring manual downloading and compilation by the user. This eliminates a common problem in bioinformatics where software may require technical expertise to first compile and install before being used. Installed plugins may also be locked to specific versions to prevent backwards incompatible software changes breaking an existing workflow.

### Integration with the Command Line

Genomer is built as a command line tool around plain text files. This allows for integration with common Linux tools tools such as GNU Make or git allowing reproducible scripting of the genome finishing process.

Genomer is written as a command-line tool for use in shells such as bash or zsh. The advantage of command-line tools over GUI-based tools, is that the former are simpler to automate and allow the sharing of scripts with other researchers working on related projects. For instance, during our microbial genome project we automated Genomer using GNU Make build files, allowing the finishing steps to be repeated automatically using the make command. Genomer may therefore be of particular interest to bioinformaticians coordinating in groups on the same genome project and those who prefer automated approaches and scripting on the command line.

As Genomer uses plain text files to manage the draft genome sequence it is easy to store versions of the project using a revision control system such as git [15]. This allows tracking changes to the project and reverting errors back to earlier versions. Example versioned build files from our genome projects can be found on GitHub for a simple plasmid [16] and a more complex genome [17] sequence.

We have released Genomer as open source software on GitHub [18] and have prepared documentation and video tutorials at http://next.gs. We have used this tool extensively to simplify our own genome projects and believe Genomer may also be useful to others in the field. We will illustrate the possible application of Genomer with the following use case.

### Genomer use Case

1. A Genomer project is organised around a set of already assembled and annotated contigs. In our microbial genome projects we assemble our reads into contigs using the A5 pipeline [19] and then subsequently annotate them using the Integrated Microbial Genomes resource [20]. We then use these contigs as a starting point to build a draft genome sequence in Genomer.
2. A genome scaffold is written in the scaffolder file format [13]. This specifies the order and orientation of contigs and unresolved gap regions in the scaffold. Paired-read sequencing or comparison to reference genomes can be used as a source of contig orientation and order. As an example, in our own genome projects we determine contig order by aligning to a reference genome using nucmer [21]. A detailed overview of the scaffolder file format can be found on the Genomer website.
3. The genomer view command is used to generate files of the assembled scaffold, such as the FASTA file of the assembly or a GFF3 file of annotations locations on the assembled genome sequence. These files can then be used for subsequent analyses of the sequence.
4. The genome scaffold can be continuously updated through closing any remaining gaps. During our microbe sequencing projects we closed gaps through a combination of Sanger PCR and *in silico* analysis. Genomer allows changes in the scaffold file to automatically propagate to the generated files when the genomer view command is rerun.
5. The required files for submission to GenBank may be generated when researchers are satisfied with the status of their draft. These files include the assembled FASTA file, contig FASTA file, annotation table file, and AGP scaffold file. Additional options can be used to add the required prefix to gene annotation IDs and begin locus ID numbering at the origin of the sequence. Genomer does not produce the.asn file which is submitted to GenBank, but instead the files that are used as the input to tbl2asn or sequin tools provided by GenBank.

### Limitations

Genomer was written to satisfy our needs for building and finishing megabase sized draft genomes where assembly of the sequence takes place in memory. This makes Genomer well-suited for microbial genome projects where approximately 99% of microbial genomes sequenced to date are less than 10 MBp. The assembly of very large sequences may however limit performance when the size of the sequence exceeds available RAM space. We however anticipate that genome sizes of even 3 GBp will fit in the memory of a modern laptop or desktop computer.

One features of Genomer is the simplification of generating the annotation table file required for submission to GenBank. Genomer requires only a GFF3 file containing gene-type entries. Genomer is then able to generate the additional encoded protein features from these entries. One caveat to this however is that the assumption there is no intron/exon structure and therefore users wishing to submit annotations with alternative splicing cannot take advantage of this Genomer feature.

### Conclusions

Genomer is an open-source tool to simplify and automate repetitive and time-consuming tasks required when finishing a microbial genome project. This software is available on GitHub [18] with documentation and video tutorials on the http://next.gs website. This tool has been useful in our own genome projects, and we believe it will also be useful to other researchers especially smaller research groups entering the field for the first time.
